# Diet-Induced Maternal Obesity Alters Insulin Signalling in Male Mice Offspring Rechallenged with a High-Fat Diet in Adulthood

**DOI:** 10.1371/journal.pone.0160184

**Published:** 2016-08-01

**Authors:** Thaís de Fante, Laís Angélica Simino, Andressa Reginato, Tanyara Baliani Payolla, Débora Cristina Gustavo Vitoréli, Monique de Souza, Márcio Alberto Torsoni, Marciane Milanski, Adriana Souza Torsoni

**Affiliations:** Laboratory of Metabolic Disorders, School of Applied Sciences, University of Campinas–UNICAMP, Limeira, São Paulo, Brazil; Universidade do Estado do Rio de Janeiro, BRAZIL

## Abstract

Modern lifestyle has resulted in an increase in the prevalence of obesity and its comorbidities in pregnant women and the young population. It has been well established that the consumption of a high-fat diet (HFD) has many direct effects on glucose metabolism. However, it is important to assess whether maternal consumption of a HFD during critical periods of development can lead to metabolic changes in the offspring metabolism. This study evaluated the potential effects of metabolic programming on the impairment of insulin signalling in recently weaned offspring from obese dams. Additionally, we investigated if early exposure to an obesogenic environment could exacerbate the impairment of glucose metabolism in adult life in response to a HFD. Swiss female mice were fed with Standard Chow (SC) or a HFD during gestation and lactation and tissues from male offspring were analysed at d28 and d82. Offspring from obese dams had greater weight gain and higher adiposity and food intake than offspring from control dams. Furthermore, they showed impairment in insulin signalling in central and peripheral tissues, which was associated with the activation of inflammatory pathways. Adipose tissue was ultimately the most affected in adult offspring after HFD rechallenge; this may have contributed to the metabolic deregulation observed. Overall, our results suggest that diet-induced maternal obesity leads to increased susceptibility to obesity and impairment of insulin signalling in offspring in early and late life that cannot be reversed by SC consumption, but can be aggravated by HFD re-exposure.

## Introduction

Worldwide, the prevalence of obesity in the younger population suggests that the intrauterine environment and lactation can contribute considerably to the development of obesity through foetal programming of offspring metabolism and energy balance [1, 2]. In experimental animals, the effect of maternal obesity on offspring metabolism has been studied mostly in models of diet-induced obesity [3]. Dietary fatty acids may influence metabolism, leading to lipid accumulation, an increase in circulating fatty acids and the development of metabolic syndrome [4]. During pregnancy and lactation, high-fat diet (HFD) has been shown to be associated with changes in lipid metabolism, increased cholesterol levels, the development of fatty liver, endoplasmic reticulum stress, impaired hypothalamic glucose metabolism, deregulation of energy homeostasis and food intake, and the deterioration of β-cell function in offspring [5, 6, 7, 8, 9, 10, 11, 12, 13]. In addition, maternal insulin resistance in the absence of obesity can impair glucose metabolism in the offspring [14].

Obesity and its comorbidities are followed by an inflammatory condition [15]. Inflammation is casually linked with impaired insulin signalling in peripheral tissues (e.g. liver, adipose and muscle), and the central nervous system (e.g. hypothalamus) [16, 17, 18]. Pro-inflammatory cytokines, mainly TNFα, can inhibit insulin signalling by targeting IRS (insulin receptor substrates) proteins or insulin receptors. TNFα and other cytokines can stimulate serine kinases, such as c-Jun N-terminal kinase (JNK) and IκB kinase (IKK), to phosphorylate IRS proteins, leading to the inactivation of proteins involved in insulin signalling [19].

Pregnancy is also characterized by an inflammatory process in the placenta. During pregnancy, consumption of a HFD may exacerbate the inflammatory response by stimulating macrophage infiltration, inflammatory signalling pathways and the production of inflammatory markers [20, 21]. In obese mothers, it has been shown that cells from the umbilical cord have alterations in gene expression that may promote inflammation and the development of metabolic syndrome in the offspring [22]. In addition, a recent study showed that the methylation pattern of genes in blastocysts was altered in the offspring of obese dams [23]. Similarly, in the offspring of obese dams after weaning, there was impaired expression of microRNAs that control fatty acid oxidation, indicating a strong influence of maternal nutritional status on epigenetic inheritance and metabolic programming [24]. In mice, exposure to a HFD during early development and consumption of a HFD after weaning increased susceptibility to the development of severe liver disease [25]. In addition, overnutrition by decrease in litter size on postnatal day 1 can lead to increased obesity susceptibility and insulin and leptin resistance after consumption of a HFD in later life [26]. Kruse and colleagues (2013) recently showed that offspring from obese dams fed with a HFD in adulthood presented with increased body weight, blood glucose and insulin levels, and liver triglycerides, compared with offspring of control dams [27]. Given the effects of obesity on metabolism and signalling pathways and the early exposure of foetuses to an inflammatory environment in the womb, we hypothesized that early contact of offspring to an inflammatory environment during gestation and lactation would increase the inflammatory response after a secondary exposure to a HFD in adult life.

In this study we evaluated the potential effect of metabolic programming in the impairment of insulin signalling in recently weaned offspring of dams fed a HFD during pregnancy and lactation as well as in adult life in response to consumption of a HFD. This manuscript brings together results of white adipose tissue, skeletal muscle, liver, hypothalamus and metabolic parameters. All analysis were performed using the same model, age and nutritional protocol in an integrated approach.

## Methods

### Animal model and diet

Swiss mice were provided by the Animal Breeding Center at the University of Campinas (Campinas, SP, Brazil) and housed in a 12 h light/dark cycle in a temperature-controlled environment. Ethics approval was obtained from the State University of Campinas Ethics Committee (Protocol 3234–1). All animal procedures followed the Guide for the Care and Use of Laboratory Animals published by National Institute of Health [28] and the guidelines of Brazilian College for Animal Experimentation. In summary, in order to prevent infections that may cause clinical diseases we applied animal biosecurity practices [28]. In general, animals were daily monitored or when needed for experimental procedures. Animals were accompanied to signs of illness, injury, or abnormal behaviour by a person trained to recognize it. Additionally, data about weight gain, behaviour, and food intake were also used for monitoring the animal’s health. During the experimental period none of the animals became sick or had to be early sacrificed.

**Fig 1:**
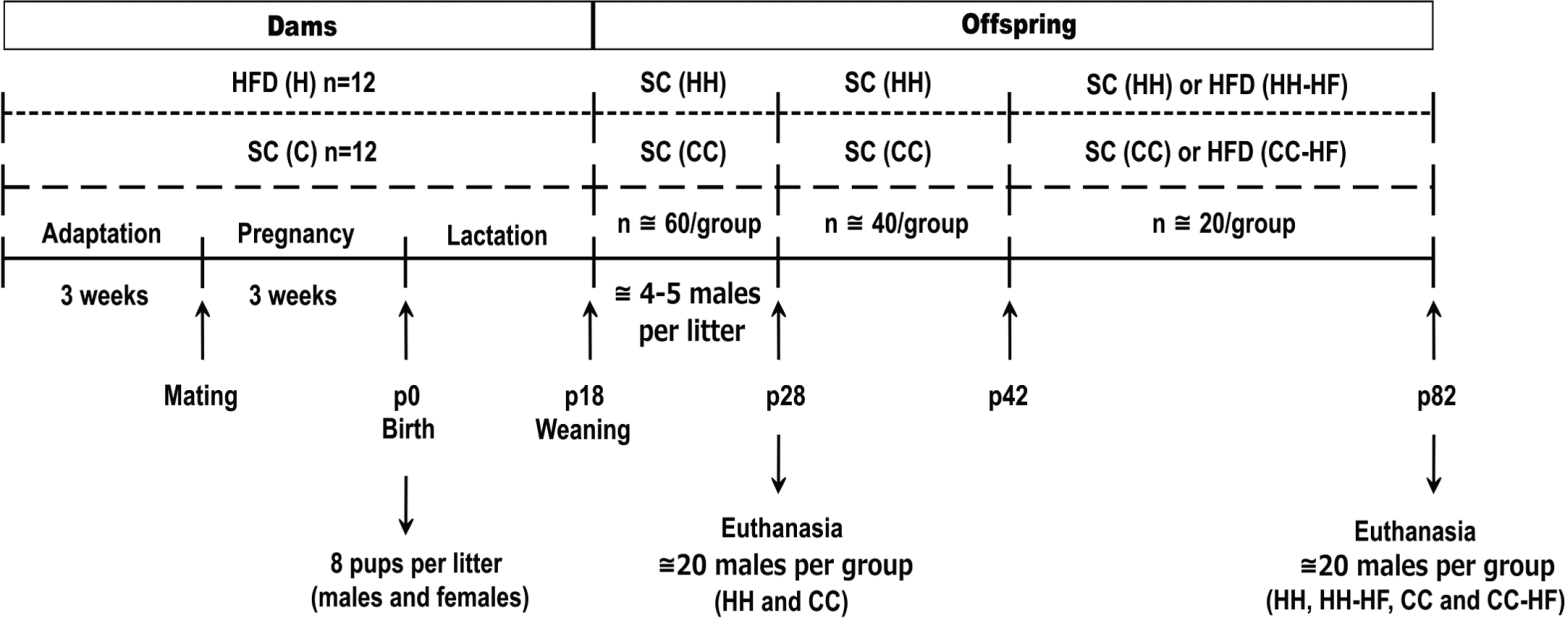
Experimental protocol of metabolic programming used in the study. Dams were fed with standard chow (SC) or a high-fat diet (HFD) during the adaptation period, pregnancy and lactation. After weaning, offspring was fed only with SC until d28 or d42. After d42, offspring from obese (H) or control dams (C) received SC (CC and HH) or HFD (CC-HF and HH-HF) until d82.

Five-week-old female mice were randomly fed *ad libitum* with SC or a HFD(G) during the adaptation period, mating, pregnancy and lactation. For experimental protocol was used twelve female per group (C or H). As previously described [9], the HFD was prepared according to the AIN-93 modified to contain a higher fat content (45%) (Table 1). After three weeks of adaptation, mating was performed with same age adult males fed only with SC. Litters were adjusted to eight pups each (containing males and females) and two groups were obtained: CC and HH, in which pups were suckled by dams exposed to SC (C) or HFD (H) during gestation and lactation, respectively. Pups were weaned on d18 and separated according to sex. Only male offspring were used in experiments. Part of the pups from CC and HH groups was maintained on SC after weaning until day 28 (d28), the first time-point. The remaining litters were maintained at SC until day 42 (d42), when part of the litters remained on standard chow diet (CC and HH) or was re-challenged with a HFD(M) until day 82 (CC-HF and HH-HF), the second time point (Fig 1).

**Table 1:** Nutritional composition used during the experimental period.

	SC*		HFD (G)**		HFD (M)***	
	g %	KJ/g	g %	KJ/g	g %	KJ/g
**Net protein**	22.5^a^	3.7^a^	20.8^b^	3.5^b^	14.9^b^	2.4^b^
**Fat content**	4.5^c^	1.7^c^	23.6^d^	8.9^d^	23.6^d^	8.9^d^
**Carbohydrates**	55.0^e^	9.2^e^	41.2^e^	6.9^e^	48.3^e^	8.0^e^
**Fibrous matter**	8.0	0	5.8	0	5.2	0
**Ash matter**	10.0	0	8.6	0	8.0	0
**Total**	100.0	14.6	100.0	19.3	100.0	19.3

* NUVILAB® Cr-1; Nuvital.

**G—Growth, offered to dams during adaptation, pregnancy and lactation periods.

***M—Maintenance, offered to offspring from d42.

a: vegetal protein—from wheat and corn (added lysine and methionine)

b: animal protein—casein

c: soy oil

d: soy oil and lard

e: starch and saccharose

### Food intake

Food intake was evaluated on d28 and d42 mice. First, mice were individually housed for three days to adapt to the environment. Following this adaptation period, the food intake was measured daily for seven consecutive days.

### Glucose Tolerance Test (GTT), Insulin Tolerance Test (ITT) and Pyruvate Tolerance Test (PTT)

Tolerance tests were performed only in adult offspring. For GTT, ITT and PTT the same animals were used, with an interval of one week between each test. Glycaemia was determined in an Accu-Chek Performa Glucometer (Roche Diagnostics, Basel, Switzerland).

For the GTT (day 63), mice were starved for 12 h, fed for 2 h and starved for an additional 4 h before intraperitoneal (IP) injection of glucose (1 g/kg of a 25% solution of D-glucose). Blood samples were collected at 0, 15, 30, 60, 90 and 120 min after glucose administration for measurement of blood glucose levels. For the ITT (day 70), a similar fasting protocol was used. Recombinant regular insulin (1,5 UI/kg) was administrated by IP injection and blood samples were collected at 0, 3, 6, 9, 12 and 15 min after injection (Humulin^®^, Eli Lilly and Company, EUA). For the PTT (day 77), IP injection of a pyruvate solution (20%) was administered (2 g/kg) in fasted mice (starved for 12 h and fed for 2 h). Blood samples were collected following the GTT protocol.

In both the GTT and the PTT, the area under curve of glycaemia *vs*. time was calculated above each individual baseline. In the ITT, the constant for the glucose disappearance rate during the test was calculated using the formula 0,693/t_1/2_.

### Hepatic glycogen

The hepatic glycogen concentration was determined as described by Dubois et al. (1956) [29]. Frozen hepatic fragments (200 mg) were digested in a 30% KOH solution for 1 h at 90°C. Subsequently, samples were saturated with Na_2_SO_4_ and then treated with 70% ethanol to precipitate glycogen. For glycogen quantification, a colorimetric method consisting of phenol and sulphuric acid was used and the absorbance was measured at 490 nm.

### Biochemical measurements

At the end of adult experimental period (d82) and after overnight fasting, mice were sacrificed. Blood samples were collected and centrifuged for 30 min at room temperature, and serum aliquots were used to measure serum TNFα and leptin. Serum TNFα was measured using an ELISA MAX^TM^ Deluxe Sets kit (BioLegend, CA, USA). To determinate serum leptin levels, a mouse Leptin ELISA kit (Sigma-Aldrich, MO, USA) was used.

### Tissue extraction and Western blot

Samples of hypothalamus, liver, soleus muscle, and epididymal adipose tissue from male offspring were collected at d28 and d82. Mice received a mixture containing ketamine (139.2 mg/kg bw), diazepam (4 mg/kg bw) and xylazine (18.4 mg/kg bw) as anesthetise protocol and decapitation was used to culled mice that were starved for 12 h. To evaluate insulin signalling, a *bolus* injection of saline or regular insulin (5 UI) through the abdominal cava vein was administered (Humulin^®^, Eli Lilly and Company, EUA). Subsequently, hepatic, soleus, adipose and hypothalamic samples were extracted after 45 s, 90 s, 3 min and 10 min, after injection respectively. To evaluate hypothalamic signalling of leptin, fasted cannulated mice from different groups received 10 μl of leptin (1 ng/μl) or saline intracerebroventricular (icv) and the hypothalamus was removed 10 to 15 min later. For either animals stimulated with insulin or leptin, was considered the delta value (value after stimulation—value before stimulation) for the statistical analysis (S1 and S2 Figs).

Tissue samples were weighted, frozen in liquid nitrogen and stored at -80°C until processing. The tissues were homogenized in freshly prepared ice-cold buffer [1% (v/v) Triton X-100, 0.1 mol/L Tris, pH 7.4, 0.1 mol/L sodium pyrophosphate, 0.1 mol/L sodium fluoride, 0.01 mol/L EDTA, 0.01 mol/L sodium vanadate, 0.002 mol/L PMSF, and 0.01 mg/mL aprotinin]. Insoluble material was removed by centrifugation (10,000 x g) for 30 min at 4°C. The protein concentration in the supernatant was determined using the Bradford dye-binding method. The supernatant was resuspended in Laemmli sample buffer and boiled for 5 min before separation by SDS-PAGE using a miniature slab gel apparatus (BioRad, Richmond, CA, USA). Electrotransfer of proteins from the gel to a nitrocellulose membrane was performed for 120 min at 120 V (constant). Nitrocellulose membranes were probed overnight at 4°C with specific antibodies as described in Table 2.

**Table 2:** Antibodies used in Western Blot analysis.

Antibody	Source	Antibody	Source
p-AKT (sc-7985)*	Rabbit polyclonal	PEPCK (sc-32879)*	Rabbit polyclonal
total AKT (sc-8312)*	Rabbit polyclonal	p-JAK2 (sc-16566-R)*	Rabbit polyclonal
p-IRS1 (sc-17200)*	Rabbit polyclonal	total JAK2 (sc-278)*	Rabbit polyclonal
total IRS1 (sc-559)*	Rabbit polyclonal	total STAT3 (sc-483)*	Rabbit polyclonal
p-JNK (sc-6254)*	Mouse monoclonal	β-actin (ab8227)**	Rabbit polyclonal
total JNK (sc-1648)*	Mouse monoclonal	PTP1B (ab52650)**	Rabbit monoclonal
p-IKK (sc-21661)*	Rabbit polyclonal	p-STAT3 (#9145)***	Rabbit monoclonal
total IKK (sc-34673)*	Goat polyclonal	α-tubulin (T5168)****	Mouse monoclonal

* Santa Cruz, CA, USA

** Abcam (Cambridge, MA, USA)

*** Cell Signaling (Danvers, MA, USA)

**** Sigma (Sigma-Aldrich, MO, USA)

Membranes were incubated for 2 hours in room temperature with HRP-conjugated secondary antibodies (KPL, Gaithersburg, MD, USA). Proteins recognized by the secondary antibodies were detected by chemiluminescence (SuperSignal West Pico Chemiluminescent Substrate, Thermo Fisher Scientific, MA, USA). The results were visualized by autoradiograph with preflashed Kodak XAR film. Band intensities were quantified by optical densitometry of developed autoradiographs using Scion Image software (ScionCorp, MD, USA) and the intensities of the bands were normalized to those of total protein or loading control (α-tubulin or β-actin).

### Quantitative Real-Time PCR (qRT-PCR)

Total epididymal white adipose tissue (WAT) RNA was extracted using TRIzol reagent (Life Technologies Corporation, CA, USA) according to the manufacturer’s recommendations and was quantified using a NanoDrop ND-2000 (Thermo Electron, WI, USA). Reverse transcription was performed with 3 ng of total RNA, using a high-capacity cDNA reverse transcription kit (Life Technologies Corporation, CA, USA). Relative expression levels were determined using a Taqman detection system and primers for the target genes (Table 3).

**Table 3:** Primers used in PCR analysis.

Primer	Code	NCBI Reference Sequence
*CIDEC*	Mm00617672_m1*	NM_001301295.1
*PPARγ*	Mm.PT.5831161924**	NM_001127330(2)
*Β-actin*	4352341E***	NM_007393.1

* Life Technologies Corporation, CA, USA.

** Integrated DNA Technologies, CA, USA.

*** Applied Biosystems, CA, USA

Each PCR reaction contained 20 ng of complementary DNA. Real-time PCR was performed on an ABI Prism 7500 Fast platform. Data were analysed using the sequence detection system 2.0.5 (Life Technologies Corporation, CA, USA) and expressed as relative values determined by the comparative threshold cycle (Ct) method (2−ΔΔCt), according to the manufacturer’s recommendations.

### Cannula implant

Mice were continually instrumented with an icv (lateral ventricle) cannula and kept under controlled temperature (22 ±1°C) and light-dark conditions (12 hours) in individual metabolic cages. Surgery was performed under anaesthesia and all efforts were made to minimize animal suffering. The cannula was implanted into the lateral cerebral ventricle at pre-established coordinates from Bregma: anteroposterior 0.34 mm, lateral 1.0 mm and depth 2.2 mm according to a previously described method [30]. The correct position of the cannula was confirmed after recovery using dipsogenic response after administration of 2 μl of 10^−6^ M angiotensin II (Sigma, MO, USA).

### Statistical analyses

Results are expressed as the mean ± SEM. Blot results were quantified by densitometry and are presented as direct band comparisons in autoradiographs. For all experiments, the animals were distributed in randomized blocks to obtain reliable and stable data. Animals from at least three or four different litters were used for all experiments, including western blot. Student’s unpaired t-tests were used to compare the differences between two groups. Two-way ANOVA were applied to compare influence of maternal diet *versus* postnatal diet. Repeated measures ANOVA was used when necessary. The statistical analyses used in each graph are specified in the respective legend. A post-hoc test (Bonferroni) was used for determining a significance level of p<0.05. Data were analyzed with GraphPad Prism 7 software (GraphPad Software Inc, USA).

## Results

### Body weight, adiposity, food intake and hepatic glycogen levels

The HFD dams (H) were heavier than controls dams (C) from adaptation (1.16-fold, p<0.0001) to lactation period (1.3-fold, p<0.0001). Fat mass at d18 (adaptation period) increased in H (1.6-fold, p<0.0001), compared to C dams. During gestation (d12) and lactation (d15) fat mass was higher in H than C dams (5-fold and 6.6-fold, p<0.0001, respectively). Despite higher adiposity in H than C dams, no increase caloric intake was similar in all evaluated periods (Table 4).

**Table 4:** Evaluation of dams in adaptation, gestation and lactation periods.

	Control Dams (C)			High-Fat Dams (H)		
	Adaptation (Mean ± SEM)	Gestation (Mean ± SEM)	Lactation (Mean ± SEM)	Adaptation (Mean ± SEM)	Gestation (Mean ± SEM)	Lactation (Mean ± SEM)
Body Weight (g)	26.98 ± 0.41^a^	31.22 ± 1.62^a^	29.57 ± 0.54^a^	33.99 ± 0.62^b^	36.33 ± 1.94^b^	38.66 ± 0.29^b^
Adiposity (%)	1.41 ± 0.09^a^	0.88 ± 0.12^a^	0.51 ± 0.08^a^	2.35 ± 0.06^b^	4.55 ± 0.37^b^	3.36 ± 0.43^b^
Caloric Intake (kcal/day)	19.93 ± 1.51	28.48 ± 1.61	23.28 ± 0.42	20.45 ± 0.80	30.75 ± 2.72	23.52 ± 0.41
Fasting Glucose (mmoL/L)	7.10 ± 0.19^a^	7.01 ±0.43^a^	4.96 ± 0.57^a^	8.26 ± 0.41^b^	8.89 ± 0.17^b^	6.82 ± 0.65^b^
Serum Insulin (pmoL/L)	110.75 ± 15.82^a^	96.42 ± 4.79	274.40 ± 66.32^a^	245.15 ± 28.54^b^	90.85 ± 4.99	664.54 ± 7.31^b^

Different letters indicate significant differences at p<0.05

**Fig 2:**
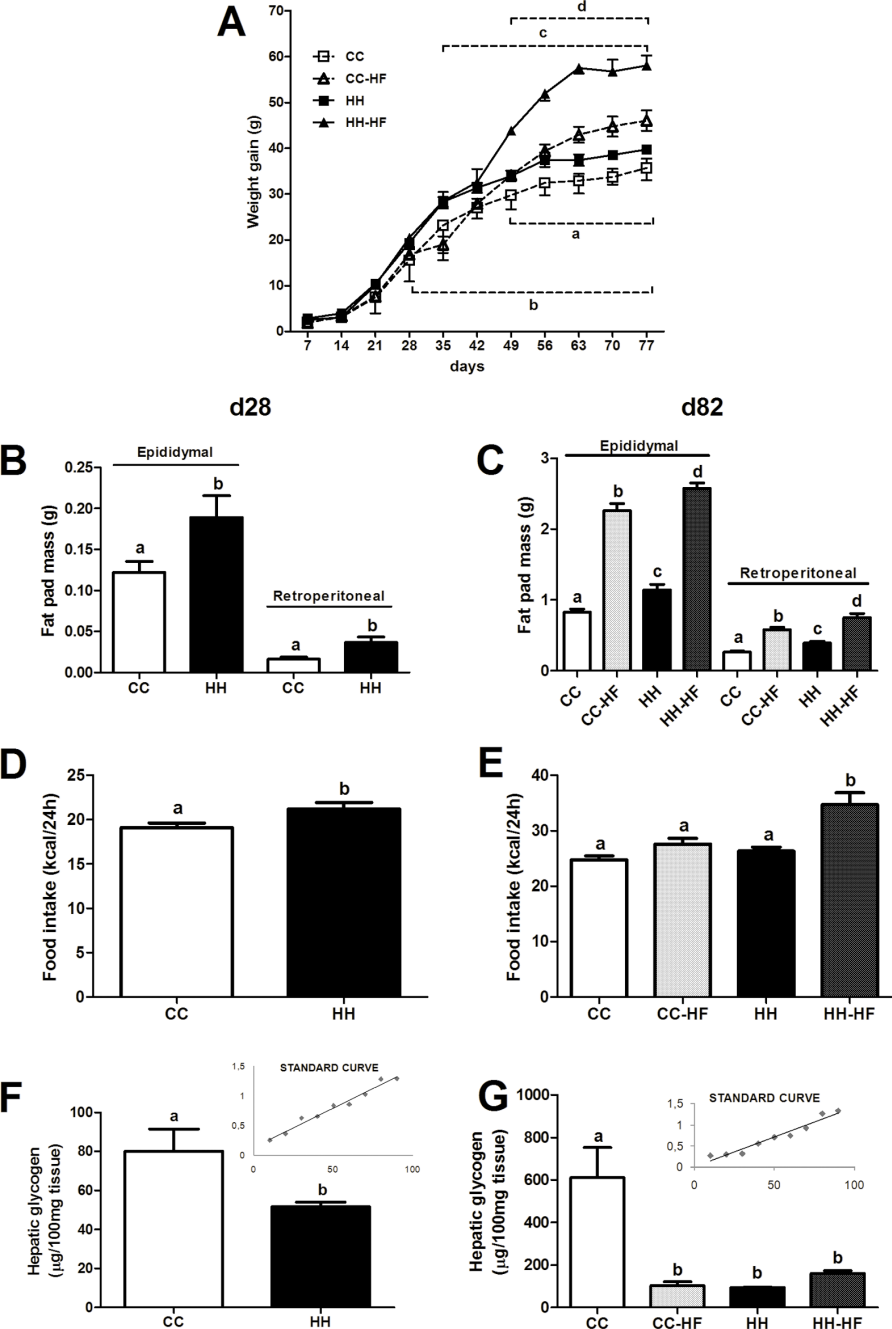
Metabolic parameters of offspring. Body weight from d7 to d77 (n = 9 per group) (A), fat pad mass at d28 (n = 19 per group) (B) and d82 (n = 21 per group) (C), food intake at d28 (n = 8 per group) (D) and d82 (n = 15 per group) (E), and hepatic glycogen at d28 (n = 10 per group) (F) and d82 (n = 3 per group) (G). Data are means ± SEM. In all graphs, at least three different litters were considered. In A, a = CC *vs*. CC-HF, b = CC *vs*. HH, c = CC-HF *vs*. HH-HF, d = HH *vs*. HH-HF. In others, different letters indicate significant differences at p<0,05. Repeated measures two-way ANOVA (A), two-way ANOVA (C, E and G) or t test (B, D and F) was used to compare the groups.

Offspring were weighed on d7 and weekly thereafter. On d28 mice from the HH group weighed 10% more (p = 0.0155) than mice from the CC group. However, in adult life consumption of a HFD (HH-HF mice) resulted in greater body weight than mice from fed a control diet (HH). Besides, body weight was 28% higher (p<0.0001) in HH-HF mice than CC-HF mice (Fig 2A). In addition, on d28, the HH group had 54% (p = 0.0370) and 127% (p = 0.0120) greater epididymal and retroperitoneal fat pad mass, respectively, and increased their food intake by 10% (p = 0.0283) (Fig 2, 2B and 2D). On d82, HH and CC mice were not different concerning food intake but HH presented higher fat pad mass (38%, p = 0.0213 for epididymal, and 48%, p = 0.0423 for retroperitoneal) compared to CC mice. On d82 after the HFD consumption period (from d42 to d82), adiposity increased in both groups, but the effect of HFD consumption was significantly greater in the HH-HF mice, 14% (p = 0.0339) and 29% (p = 0.0196) in epididymal and retroperitoneal fat, respectively (Fig 2C). Food intake measurements on d82 also suggested that food intake was affected by maternal diet. As showed in the Fig 2E, HH-HF mice had 25% higher (p = 0.0001) food intake than CC-HF mice. On d28, hepatic glycogen levels were 35% lower (p = 0.0214) in the HH group compared with CC group (Fig 2F). On d82, CC-HF (p = 0.0064), HH (p = 0.0058), and HH-HF (p = 0.0131) groups had lower hepatic glycogen levels (less than 70%) compared with that of the CC group (Fig 2G).

### Serum biochemical parameters

Fasting glucose and insulin levels of dams were analyzed in the adaptation (d18), gestation (d12) and lactation (d15) period. As can observed in the Table 4, fasting glucose and insulin levels were affected by HFD consumption.

**Fig 3:**
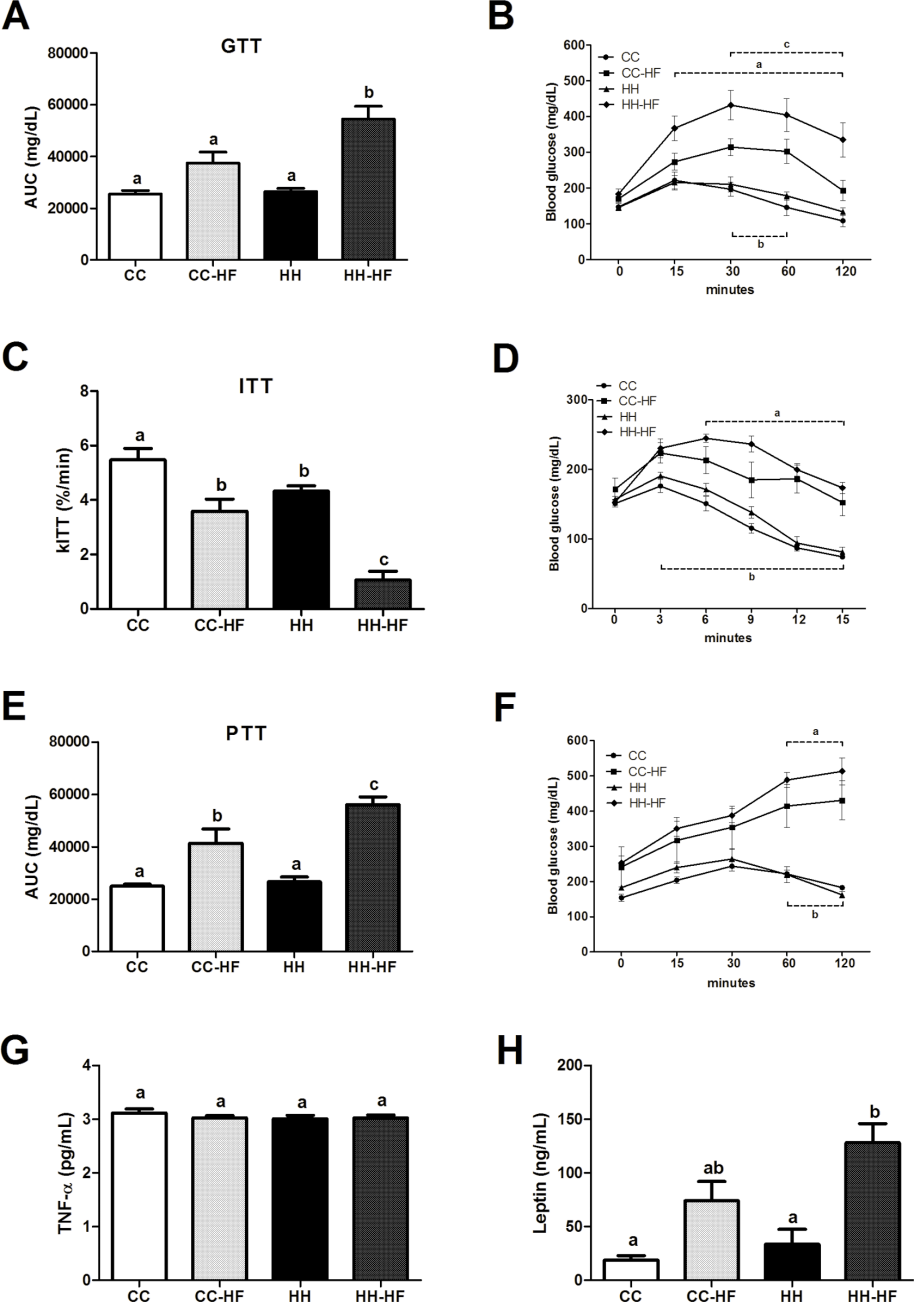
Serum biochemical parameters and glucose homeostasis. Glucose tolerance test at d63 (n = 6 per group) (A and B), insulin tolerance test at d70 (n = 6 per group) (C and D), pyruvate tolerance test at d77 (n = 6 per group) (E and F), serum TNF-α at d82 (n = 4 per group) (G) and serum leptin at d82 (n = 4 per group) (H). Data are means ± SEM, repeated measures two-way ANOVA (B, D, F) or two-way ANOVA (A, C, E, G and H) was used. In all analysis, at least three different litters were considered. In B, D and F, a = HH vs. HH-HF, b = CC vs. CC-HF, c = CC-HF vs. HH-HF. In others, different letters indicate significant differences at p<0.05.

Intraperitoneal ITT, GTT and PTT were performed in adult offspring (Fig 3). Maternal consumption of HFD did not alter glucose tolerance or liver glucose production (PTT) in adult offspring fed with a control diet. However, as expected, adult offspring mice fed HFD had glucose and insulin intolerance and increased glucose levels (Fig 3A–3F); however, the effect was greater in offspring from obese dams than control dams. HH-HF showed increased area under curve in GTT (45%, p = 0.0180) and PTT (36%, p = 0.0138), and 70% decrease in kITT (p = 0.0015). Serum TNF-α levels were not different, but serum leptin levels were higher in HH-HF mice compared to CC (6.7-fold, p = 0.0043) and HH (3.8-fold, p = 0.0106), and showed a tendency to increase 1.7-fold (p = 0.1803) in HH-HF compared with CC-HF mice as shown in Fig 3G and 3H.

### Expression and activation of inflammatory markers and insulin and leptin signalling proteins in newly weaned mice (d28)

**Fig 4:**
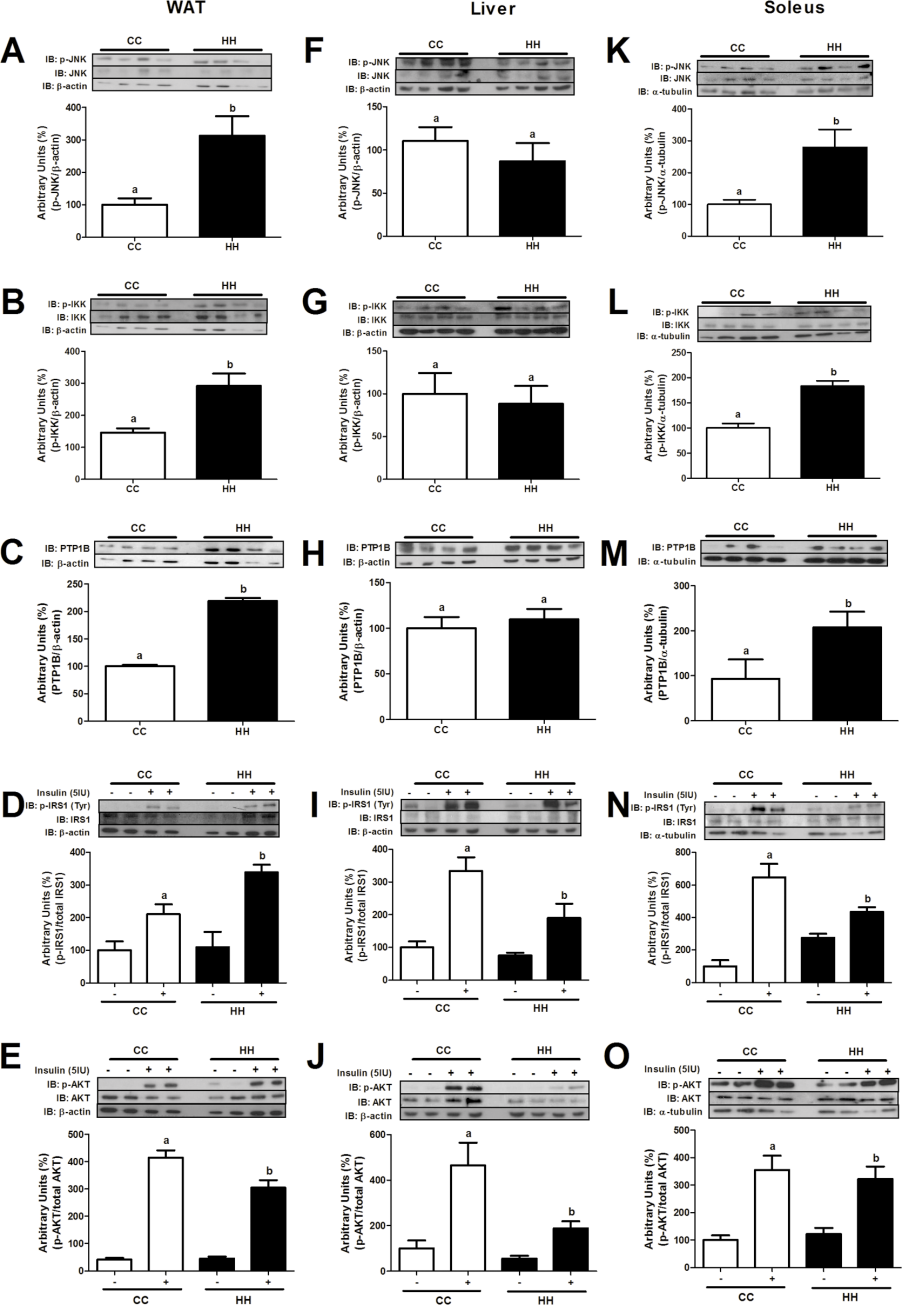
Inflammatory and insulin signalling proteins in peripheral tissues at d28. Western blot analysis of p-JNK (A), p-IKK (B), PTP1B (C), p-IRS1 (D), and p-AKT (E) in WAT, p-JNK (F), p-IKK (G), PTP1B (H), p-IRS1 (I), and p-AKT (J) in liver, p-JNK (K), p-IKK (L), PTP1B (M), p-IRS1 (N), and p-AKT (O) in soleus at d28. For control of gel loading, membranes were reblotted with β-actin, α-tubulin or total proteins. Data are means ± SEM (n = 3–8). T test analysis was used. In all blots, at least three different litters were considered. Different letters indicate significant differences at p<0.05.

**Fig 5:**
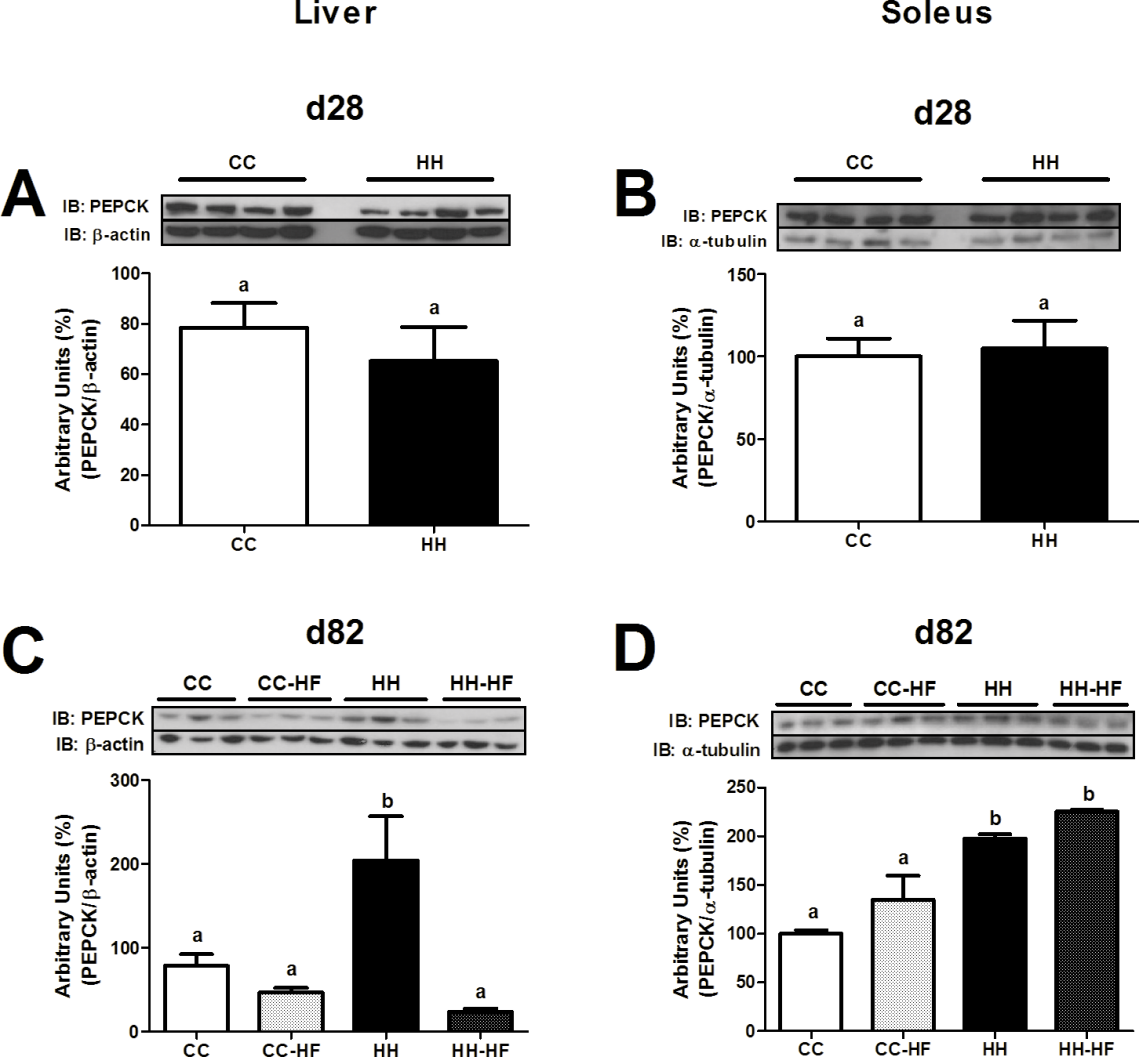
PEPCK content in liver and soleus. Western blot analysis of PEPCK in liver at d28 (A) and d82 (C), in soleus at d28 (B) and d82 (D). For control of gel loading, membranes were reblotted with β-actin or α-tubulin. Data are means ± SEM (n = 3–8). Two-way ANOVA (C and D) or t test (A and B) was used. In all blots, at least three different litters were considered. Different letters indicate significant differences at p<0.05.

To evaluate insulin resistance and the activation of inflammatory pathways in the hypothalamus and peripheral tissues in newly weaned offspring (d28), Western blot analysis was performed. As shown in Fig 4, WAT from HH mice had greater JNK and IKK phosphorylation (3.1-fold, p = 0.0291, and 2.0-fold, p = 0.0118, respectively), PTP1B expression (2.2-fold, p<0.0001), and reduced AKT phosphorylation (p = 0.0001) compared with CC mice (Fig 4A–4E). In the liver, there were no differences in the phosphorylation status of JNK, IKK (Fig 4F and 4G) and PTP1B (Fig 4H) and PEPCK expression levels remained unchanged (Fig 5A). On the other hand, insulin-stimulated IRS-1 (p = 0.0456) and AKT (p = 0.0489) phosphorylation levels were 50% diminished in the HH group compared with the CC group (Fig 4I and 4J). In soleus, there was increased expression of inflammatory markers (p-JNK, 2.8-fold, p = 0.0217, and p-IKK, 1.8-fold, p = 0.0024, Fig 4K and 4L) and PTP1B (2.2-fold, p = 0.0453, Fig 4M) in the HH group compared with the CC group. Furthermore, IRS-1 and AKT phosphorylation were reduced (1.7-fold, p = 0.0288, and 1.3-fold, p = 0.0368, respectively) in the HH group compared with the CC group (Fig 4N and 4O).

**Fig 6:**
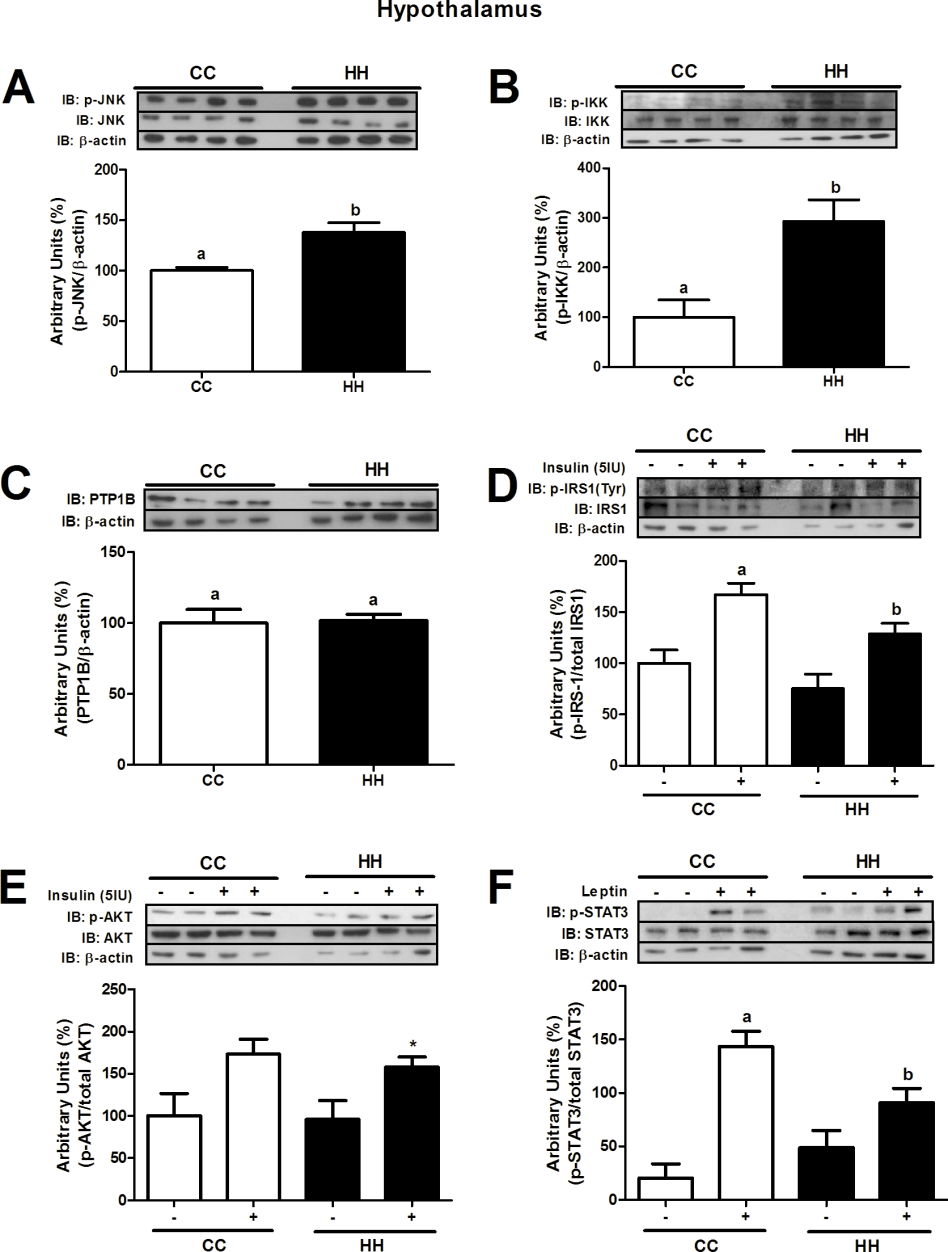
Inflammatory and insulin signalling proteins in hypothalamus at d28. Western blot analysis of p-JNK (A), p-IKK (B), PTP1B (C), p-IRS1 (D), p-AKT (E), and p-STAT3 (F) in the hypothalamus at d28. For control of gel loading, membranes were reblotted with β-actin or total proteins. Data are means ± SEM (n = 3–8). T test analysis was used. In all blots, at least three different litters were considered. Different letters indicate significant differences at p<0.05.

In the hypothalamus, the phosphorylation levels of inflammatory markers were also higher in the HH group (JNK, 1.2-fold, p = 0.0210 and IKK, 2.5-fold, p = 0.0259, respectively) than the CC group (Fig 6A and 6B). Hypothalamic PTP1B expression was the same in both groups (Fig 6C). Insulin-stimulated IRS-1 and AKT phosphorylation levels (1.4-fold, p = 0.0226, and 1.5-fold, p = 0.0039, respectively) were lower in the HH group than the CC group (Fig 6D and 6E). Furthermore, leptin-stimulated hypothalamic STAT3 phosphorylation was lower in the HH group (1.5-fold, p = 0.0071) than CC group (Fig 6F).

### Expression and activation of inflammatory markers and insulin signalling proteins in adult offspring mice after HFD consumption (d82)

**Fig 7:**
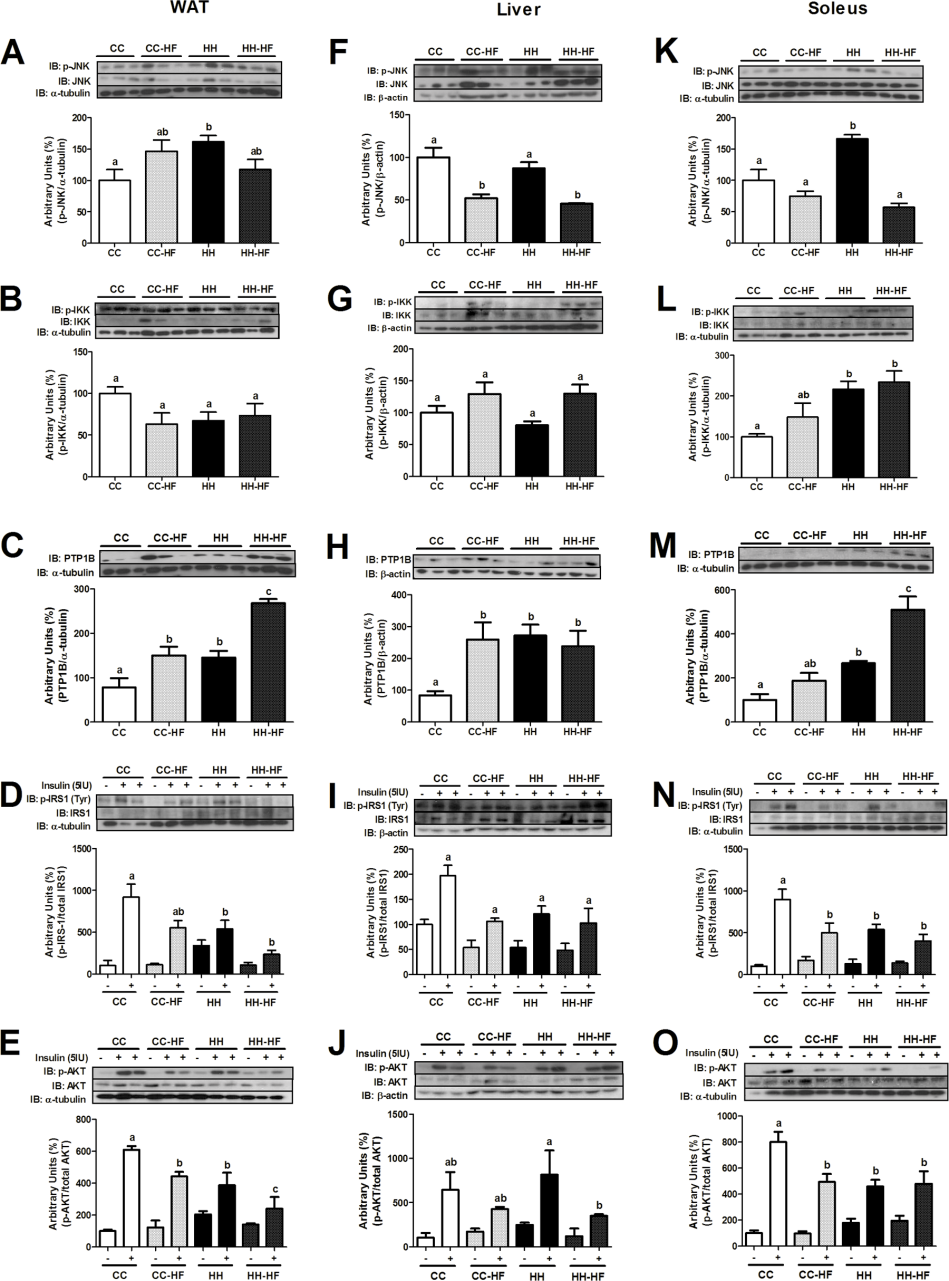
Inflammatory and insulin signalling proteins in peripheral tissues at d82. Western blot analysis of p-JNK (A), p-IKK (B), PTP1B (C), p-IRS1 (D), and p-AKT (E) in WAT, p-JNK (F), p-IKK (G), PTP1B (H), p-IRS1 (I), and p-AKT (J) in liver, p-JNK (K), p-IKK (L), PTP1B (M), p-IRS1 (N), and p-AKT (O) in soleus at d82. For control of gel loading, membranes were reblotted with β-actin, α-tubulin or total proteins. Data are means ± SEM (n = 3–8). Two-way ANOVA was used. In all blots, at least three different litters were considered. Different letters indicate significant differences at p<0.05.

JNK and IKK phosphorylation in WAT, liver, and soleus were investigated in adult offspring mice from obese (HH) and control dams (CC). In WAT and soleus, JNK phosphorylation levels were 60% higher (p = 0.0293 and p = 0.0041) in the HH group than CC group (Fig 7A–7K). However, no difference was observed in the liver (Fig 7F). p-JNK levels were not increased by HFD consumption in adult life. CC-HF and HH-HF mice showed similar JNK phosphorylation levels in WAT, liver, and soleus (Fig 7A, 7F and 7K). Similar results were observed when p-IKK levels were evaluated in WAT and liver (Fig 7B and 7G). However, maternal consumption of HFD during pregnancy and lactation was harmful to adult mice. As shown in Fig 7L, soleus p-IKK levels were higher (116%, p = 0.0495) in the HH group than the CC group.

To evaluate the effect of obesogenic environment on insulin resistance development, we evaluated PTP1B expression and IRS1 and AKT phosphorylation after insulin injection (cava vein). As shown in Fig 7C, 7H and 7M, maternal consumption of a HFD significantly increased PTP1B expression in WAT (85%, p = 0.0413), liver (220%, p = 0.0130), and soleus (166%, p = 0.0270) in the adult mice offspring. In addition, PTP1B expression levels in WAT and soleus from adult offspring fed a HFD, were higher when their mothers were obese than normal weight (control), 78% (p = 0.0029) and 171% (p = 0.0006) respectively (Fig 7C–7M). p-IRS1 and p-AKT levels in WAT, liver, and soleus were also evaluated after IP injection of insulin. As shown in Fig 7D, 7E, 7N and 7O maternal consumption of a HFD during pregnancy and lactation significantly reduced insulin-stimulated phosphorylation of IRS1 and AKT in adult offspring in WAT (84%, p = 0.0264, and 54%, p = 0.0341) and soleus (49%, p = 0.0228, and 56%, p = 0.0078) (CC *vs*. HH). Liver and soleus PEPCK expression was 97% higher (p = 0.0132 and p = 0.0030) in the HH than the CC mice (Fig 5C and 5D). In addition, p-IRS1 and p-AKT levels were also evaluated in adult offspring after HFD consumption. As shown in Fig 7E, insulin-stimulated phosphorylation of AKT was lower (52%, p = 0.0206) in WAT from the HH-HF than CC-HF mice. However, no differences were observed in liver and soleus.

**Fig 8:**
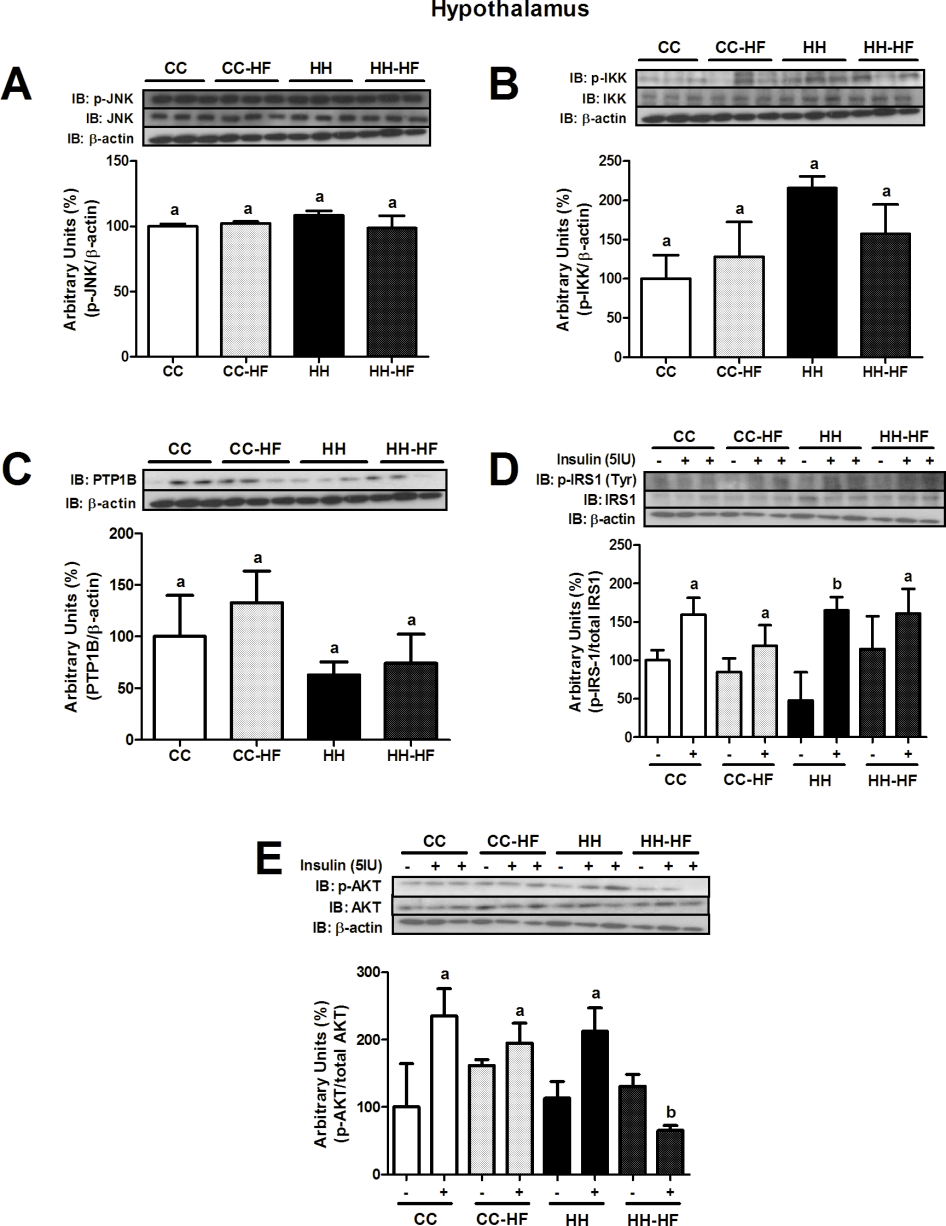
Inflammatory and insulin signalling proteins in hypothalamus at d82. Western blot analysis of p-JNK (A), p-IKK (B), PTP1B (C), p-IRS1 (D) and p-AKT (E) in the hypothalamus at d82. For control of gel loading, membranes were reblotted with β-actin or total proteins. Data are means ± SEM (n = 3–8). Two-way ANOVA was used. In all blots, at least three different litters were considered. Different letters indicate significant differences at p<0.05.

In adult mice, hypothalamic PTP1B expression and JNK, IKK, and IRS1 phosphorylation were not different between groups (Fig 8A–8D). However, hypothalamic insulin-stimulated AKT phosphorylation was significantly lower (p = 0.0078) in the HH-HF than CC-HF mice (Fig 8E).

**Fig 9:**
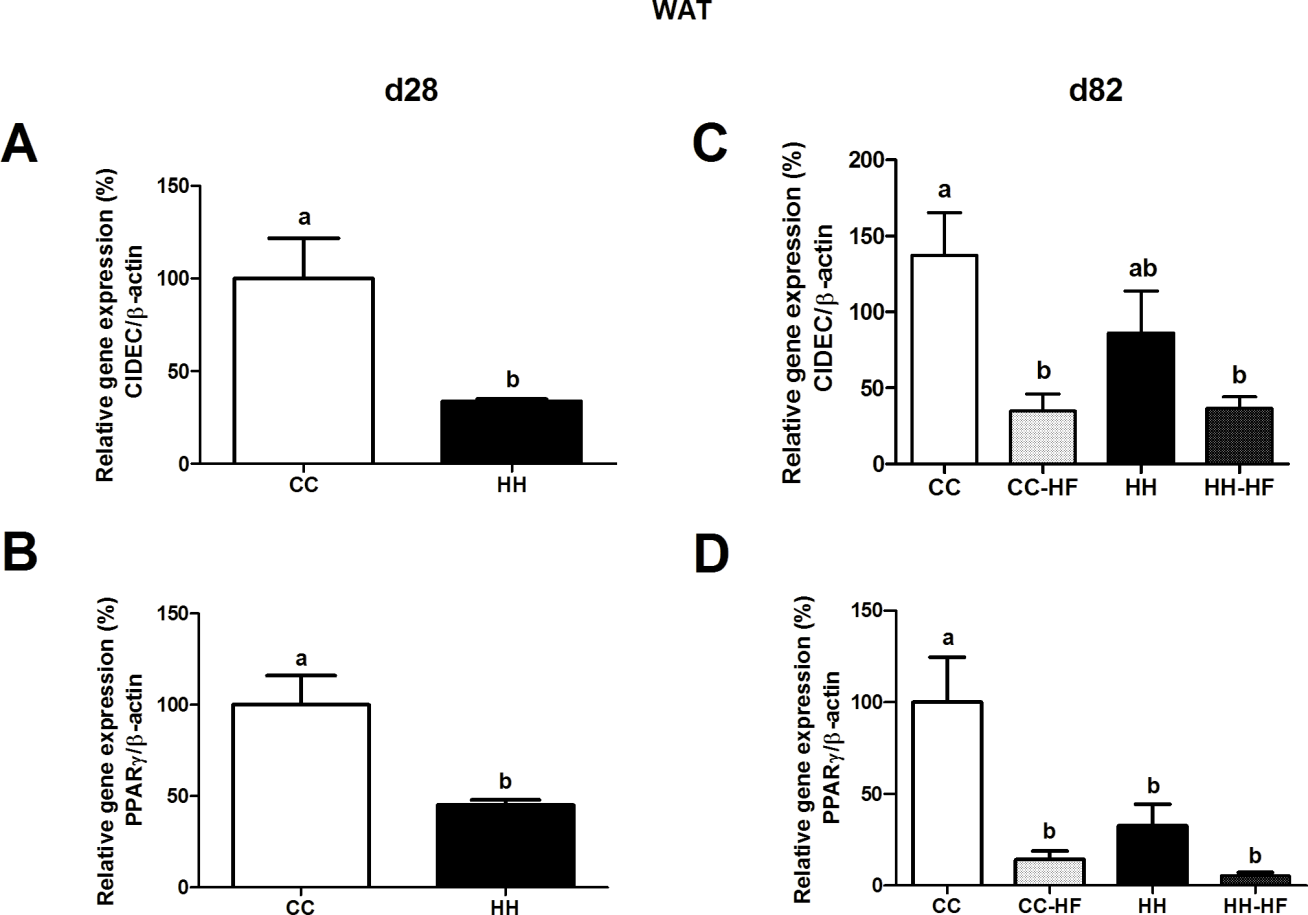
*CIDEC* and *PPARy* expression in WAT. mRNA levels (qRT-PCR) of *CIDEC* in white adipose tissue of offspring at d28 (A) and d82 (B), and *PPARy* at d28 (C) and d82 (D). For relative gene expression analysis, β-actin was used as an endogenous control. Data are means ± SEM (n = 3–8). Two-way ANOVA (C and D) or t test (A and B) was used. In all analysis, at least three different litters were considered. Different letters indicate significant differences at p<0.05.

Additionally, we evaluated *CIDEC*and *PPARγ* expression in WAT using real-time PCR (Fig 9). As shown in Fig 9A and 9B, maternal HFD consumption reduced *CIDEC* and *PPARγ* expression (66%, p = 0.0151, and 54%, p = 0.0073, respectively) in HH mice compared to CC at d28. In the adult offspring, *CIDEC* expression in HH mice returned to control levels (CC). HFD consumption during adult life significantly reduced *CIDEC* expression both in CC-HF (75%, p = 0.0402) and HH-HF (73%, p = 0.0333) compared to CC (Fig 9B). In addition, *PPARγ* expression was diminished in CC-HF (86%, p = 0.0092), HH (67%, p = 0.0282), and HH-HF (95%, p = 0.0015) mice compared with CC mice in adult life (Fig 9D).

## Discussion

Maternal nutrition has an important role in the metabolic changes observed in the offspring [3]. Obesity and activation of inflammatory pathways in the placenta have been shown to be associated with perinatal alteration in the metabolism of the offspring [7, 9, 20, 23, 24]. Initially, to validate the model of metabolic programming we evaluated body parameters in dams and offspring at d28. As expected dams fed HFD presented obese phenotype before, during and after pregnancy. As described previously [9, 24], d28 mice from obese mothers (HH) had higher body weight, epididymal and retroperitoneal fat, greater food intake, and lower hepatic glycogen levels compared with mice from control mothers (CC group). To evaluate the possibility of metabolic programming CC and HH mice were investigated in adult life. Interestingly, food intake, GTT, PTT, and leptin were not different between groups. However, the introduction of a HFD from d42 to d82 (CC-HF and HH-HF mice) resulted in higher fat pad mass, food intake, glucose and insulin intolerance and glucose production in the offspring from obese dams compared to offspring from control dams. Kruse *et al*. (2013) also described similar results in mice after HFD consumption in adult life [27]. The authors showed that diet-induced maternal obesity resulted in offspring prone to metabolic disturbances that included larger adipocytes, glucose intolerance, and insulin resistance. It is important to highlight that HFD consumption by CC-HF mice resulted in increased body weight and fat mass, but the effect of HFD consumption was greater in HH-HF mice. Increased fat mass was likely the result of increased caloric intake, as observed in HH-HF mice compared with CC-HF mice. Diet-induced maternal obesity during pregnancy seemed to be related to fat preference and proliferation of hypothalamic neurons that increase the risk for overeating and obesity [31], the same hyperphagic behavior and significant weight gain observed in our model. Neurogenesis and the establishment of new neuronal connections is occurs during pregnancy and lactation. Hormones and nutrients that influence satiety can modulate them. As suggested by the Leibowitz lab (2008), hypothalamic neuropeptides that also participate in fat preference can be modulated by maternal HFD consumption leading to harmful feeding behaviour [31]. Thus, maternal HFD consumption seems to program the offspring to have a metabolic response to HFD consumption that favours obesity.

Other studies have suggested that inflammation commonly observed in obesity models, particularly those associated with HFD consumption, is involved in hormonal resistance and obesity [9, 32, 33]. Macrophages infiltrating metabolically relevant tissues are identified as the central cellular players in metabolic inflammation, inducing alterations mediated by proinflammatory cytokines [34, 35].

Furthermore, a previous study demonstrated that offspring of dams fed with a HFD during pregnancy and lactation developed hepatic steatosis, a marker of metabolic disturbances [7]. Other authors also described reduced insulin sensitivity and altered expression of proteins related to lipid metabolism [24, 36]. Here we showed that the introduction of a HFD in adult life (from d42 to d82) in offspring resulted in the impairment of glucose homeostasis as evaluated by GTT and ITT; however, the effects were more harmful in offspring from dams fed with a HFD during pregnancy and lactation than control dams fed SC. These results could be explained by reduced phosphorylation of AKT in WAT and soleus after insulin challenge. Although the peripheral insulin resistance may be related to higher adiposity observed in HH-HF mice than CC-HF, the presence of insulin resistance in WAT and soleus of offspring of obese dams that was not challenged in adult life (HH) reinforce the effect maternal exposure to HFD on the glucose homeostasis. Furthermore, PTP1B expression was higher in the HH-HF group compared with the CC-HF group in both WAT and soleus. Studies in rodents and humans showed that higher PTP1B expression in peripheral tissues was associated with an attenuation of insulin signalling, which contributed to insulin resistance [37]. Interestingly, the endoplasmic reticulum stress and inflammation seemed to be linked with the stimulation of PTP1B expression in liver, soleus, and WAT [38, 39]. Melo *et al*. (2014) showed that recently weaned offspring had hypothalamic and hepatic activation of proteins related to protein unfolding and endoplasmic reticulum stress [9].

Leptin has an important anorexigenic effect in rodent and humans. However, although leptin levels were not higher in the HH group compared with the CC group, we observed a tendency to increase, suggesting leptin level was altered. In fact, hypothalamic leptin icv injection did not induce STAT3 phosphorylation in the HH group compared with the CC group, suggesting the presence of leptin resistance at d28. Interestingly, although we did not analyse hypothalamic p-STAT3 levels in adult mice (d82), food intake was higher and leptin levels showed a tendency to be greater in mice reexposed to HFD, indicating uncoupling between leptin levels and food intake in the offspring of obese dams. During hypothalamus development, leptin signalling establishes important new neuronal connections and lactation is a critical period for hypothalamic neurogenesis [40, 41]. Since leptin resistance was observed at d28, we not discharged the contribution of this non-physiological phenomenon to eating disorders observed. The molecular complexity of diseases associated with obesity makes it difficult to understand the effect of nutrients, hormones and epigenetic changes on the development of metabolic disorders in adult life. However, one week after weaning, offspring from obese dams weighed more than control mice, and HFD consumption promoted body weight gain, suggesting that metabolic responses to a HFD were impaired in these mice. These results reinforce the hypothesis that diet-induced maternal obesity induces permanent modifications in the offspring. On the other hand, Kang and colleagues [42] found that female offspring of obese dams exhibited increased brain microglial activation, anxiety and decreased sociability behavior but maternal dietary intervention during lactation was sufficient to alleviate social deficits and brain inflammation in females. Thus offspring disorders will occur if any intervention is performed in early life.

As previously observed by Benatti *et al*. (2014) and Melo *et al*. (2014) recently weaned offspring of obese dams had reduced insulin sensitivity, a phenomenon that has been shown to be associated with an increase in WAT and the activation of inflammatory pathways [9, 19, 24]. Interestingly, in the offspring of obese dams, the consumption of a control diet after weaning did not improve insulin-stimulated IRS-1 and AKT phosphorylation in adult mice. JNK and IKK activation and PTP1B expression in peripheral tissues reduced the effect of insulin in HH mice. Additionally, *CIDEC* expression in WAT was reduced in recently weaned HH mice and in adult mice after HFD consumption (CC-HF and HH-HF). Since *PPARγ* is essential for the transcriptional activity of *CIDEC* during adipogenesis, a reduction in *PPARγ* expression was also expected. Matsusue (2010) suggested that *CIDEC* participated in the deposition of triglycerides in adipose tissue, preventing the harmful effect of free fatty acids [43]. Furthermore, Shamsi et al. (2014) showed that in mice fed a HFD, *PPARγ* and *CIDEC* expression were reduced in visceral adipose tissue during the later phase of obesity, after gathering a large number of lipid droplets and the beginning of the lipolysis process, concomitantly to observation of insulin resistance [44]. Interestingly, in offspring, *CIDEC* expression seemed to be not influenced by maternal diet, but only for HFD consumption in adult life. Consumption of a control diet after weaning improved *CIDEC* expression in WAT, indicating a transitory effect of maternal consumption of a HFD on the offspring metabolism. However, we did not observe improve of *PPARγ* expression and insulin signalling, suggesting that metabolic imprint affected the activity of this pathway. Marco et al. (2014) studied the offspring of HFD-fed dams and found that weight and the CpG methylation status of the *Pomc* promoter were not "reprogrammed" by a control diet in rats [45].

Hypothalamic insulin and leptin signalling have important roles in peripheral metabolism [46, 47]. Hormones such as leptin, thyroid hormones, and insulin, can modulate food intake, energy expenditure, and peripheral metabolism [48]. Furthermore, several authors found that hypothalamic inflammation and insulin resistance were associated with metabolic disorders in obese animal models [49, 50, 51]. In our study, maternal consumption of a HFD led to an increase in hypothalamic JNK and IKK phosphorylation and reduced AKT phosphorylation in recently weaned offspring (d28) (Fig 6). In adult life, hypothalamic p-JNK, p-IKK, p-IRS1 and p-AKT levels were similar, but the consumption of a HFD reduced the capacity of insulin to stimulate AKT phosphorylation, without affecting p-IRS1 levels (Fig 8), even when insulin levels are increased [7]. Interestingly, hypothalamic PTP1B expression was reduced in adult offspring from dams fed with a HFD, indicating that there are other mechanisms that lead to impaired insulin action in the hypothalamus. On the other hand, the reduction of hypothalamic STAT3 phosphorylation levels in young mice indicated that there was leptin resistance after weaning that could remain until adult life. SOCS-3 is a leptin-inducible inhibitor of leptin signalling and a potential mediator of leptin resistance in obesity [52, 53]. Although, hypothalamic SOCS-3 expression was not investigated, Page and colleagues (2009) found a significant increase in SOCS-3 expression in offspring from HFD-fed dams compared with controls dams [54]. As a consequence of a high supply of nutrients to the foetus during development, hormonal resistance and damage to central mechanisms that participate in energy balance could be observed.

Thus our results emphasize the effect of overnutrition during important periods of development with an increase in the risk of adult-onset ill health outcomes, by increasing susceptibility to new stressful insults. To ensure the relevance of data, critical points during the experimental protocol were carried out, such as the efficient induction of obesity in mothers fed with HFD, litter size adjustment to ensure equal access to breast milk, the standardization of male offspring for all experiments, and the representativeness of litters. However, to ensure the number of puppies in each protocol was necessary to use more than one puppy from the same litter in each sampling. This is a limitation in the study regarding the sampling of puppies, but we took care to use in each protocol male puppies from at least three or four different litters to guarantee the heterogeneity of sampling. Overall, we conclude that the response of offspring to maternal obesity is quite homogeneous and lead to significant changes in offspring metabolism.

The major contributions of this manuscript are the deleterious effects of diet-induced maternal obesity in adult life of offspring, such as preference for high fat diet, high susceptibility to body weigh gain, and increase of fat depot when mice were challenge with high fat diet. These damages were accompanied by impairment of inflammatory parameters (p-IKK level) and insulin signaling resistance (p-AKT level) especially in peripheral tissues.

## Supporting Information

S1 Fig Delta analysis of insulin signalling proteins at d28. Delta of insulin stimulated to non stimulated p-IRS1 (A) and p-AKT (C) in WAT, p-IRS1 (B) and p-AKT (D) in liver, p-IRS1 (E) and p-AKT (G) soleus, p-IRS1 (F), p-AKT (H) and p-STAT3 (I) in the hypothalamus at d28. Data are means ± SEM (n = 3–8). T test analysis was used. Different letters indicate significant differences at p<0.05.
(TIF)

S2 Fig Delta analysis of insulin signalling proteins at d82. Delta of insulin stimulated to non stimulated p-IRS1 (A) and p-AKT (C) in WAT, p-IRS1 (B) and p-AKT (D) in liver, p-IRS1 (E) and p-AKT (G) soleus, p-IRS1 (F) and p-AKT (H) in the hypothalamus at d82. Data are means ± SEM (n = 3–8). Two-way ANOVA was used. Different letters indicate significant differences at p<0.05.
(TIF)
