# Supplementary figures and images for: Diet-Induced Maternal Obesity Alters Insulin Signalling in Male Mice Offspring Rechallenged with a High-Fat Diet in Adulthood
Source: PLoS One. 2016 Aug 1;11(8):e0160184. doi: 10.1371/journal.pone.0160184 (PMC4968809; doi:10.1371/journal.pone.0160184)

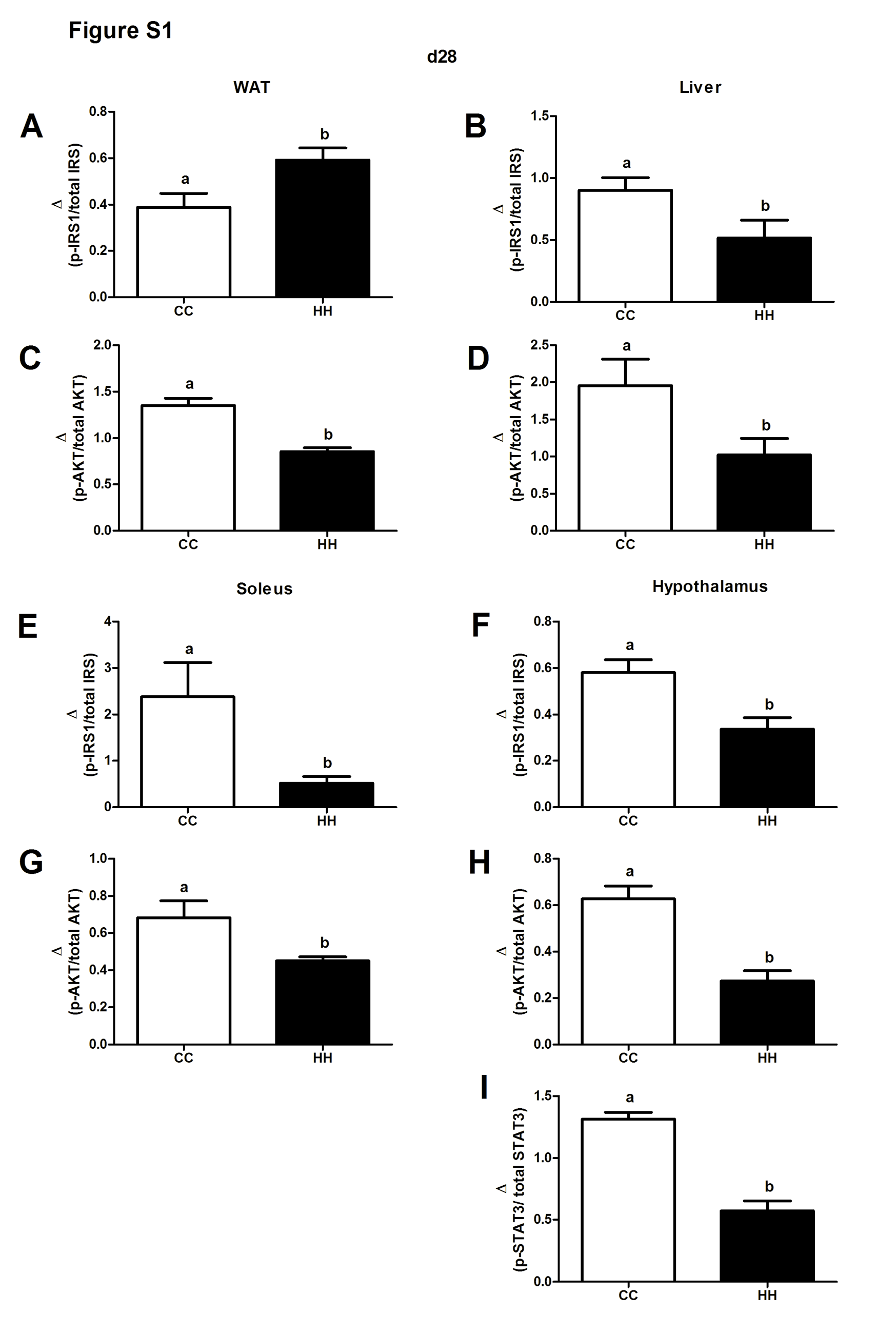

Supplement: S1 Fig — Delta of insulin stimulated to non stimulated p-IRS1 (A) and p-AKT (C) in WAT, p-IRS1 (B) and p-AKT (D) in liver, p-IRS1 (E) and p-AKT (G) soleus, p-IRS1 (F), p-AKT (H) and p-STAT3 (I) in the hypothalamus at d28. Data are means ± SEM (n = 3–8). T test analysis was used. Different letters indicate significant differences at p<0.05. (TIF) [file pone.0160184.s001.tif]

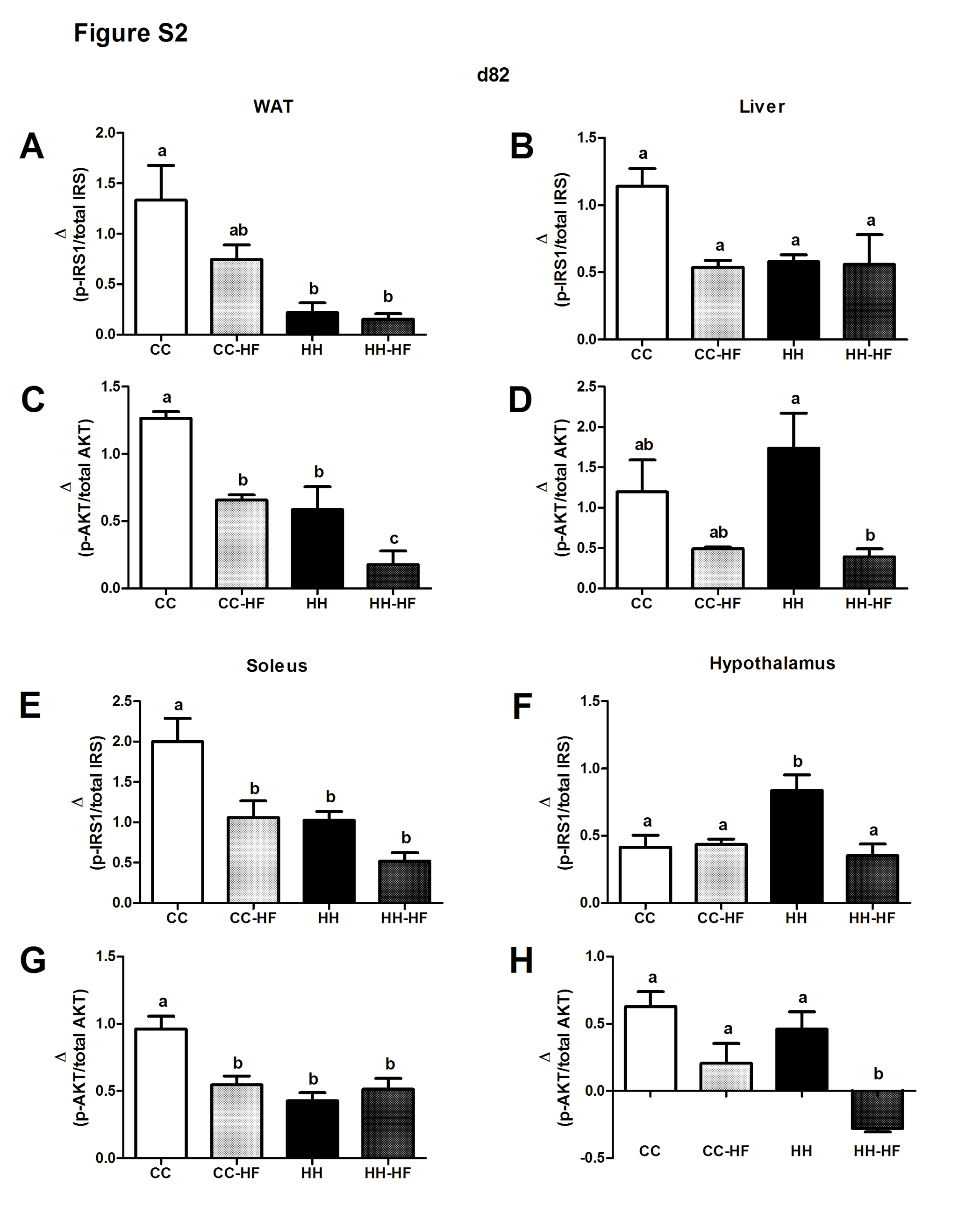

Supplement: S2 Fig — Delta of insulin stimulated to non stimulated p-IRS1 (A) and p-AKT (C) in WAT, p-IRS1 (B) and p-AKT (D) in liver, p-IRS1 (E) and p-AKT (G) soleus, p-IRS1 (F) and p-AKT (H) in the hypothalamus at d82. Data are means ± SEM (n = 3–8). Two-way ANOVA was used. Different letters indicate significant differences at p<0.05. (TIF) [file pone.0160184.s002.tif]
